# Engineered Promoters for Potent Transient Overexpression

**DOI:** 10.1371/journal.pone.0148918

**Published:** 2016-02-12

**Authors:** Dan Y. Even, Adi Kedmi, Shani Basch-Barzilay, Diana Ideses, Ravid Tikotzki, Hila Shir-Shapira, Orit Shefi, Tamar Juven-Gershon

**Affiliations:** 1 The Mina and Everard Goodman Faculty of Life Sciences, Bar-Ilan University, Ramat-Gan, 5290002, Israel; 2 Faculty of Engineering and Bar-Ilan Institute of Nanotechnologies and Advanced Materials, Bar-Ilan University, Ramat-Gan, 5290002, Israel; Weizmann Institute of Science, ISRAEL

## Abstract

The core promoter, which is generally defined as the region to which RNA Polymerase II is recruited to initiate transcription, plays a pivotal role in the regulation of gene expression. The core promoter consists of different combinations of several short DNA sequences, termed core promoter elements or motifs, which confer specific functional properties to each promoter. Earlier studies that examined the ability to modulate gene expression levels via the core promoter, led to the design of strong synthetic core promoters, which combine different core elements into a single core promoter. Here, we designed a new core promoter, termed super core promoter 3 (SCP3), which combines four core promoter elements (the TATA box, Inr, MTE and DPE) into a single promoter that drives prolonged and potent gene expression. We analyzed the effect of core promoter architecture on the temporal dynamics of reporter gene expression by engineering *EGFP* expression vectors that are driven by distinct core promoters. We used live cell imaging and flow cytometric analyses in different human cell lines to demonstrate that SCPs, particularly the novel SCP3, drive unusually strong long-term *EGFP* expression. Importantly, this is the first demonstration of long-term expression in transiently transfected mammalian cells, indicating that engineered core promoters can provide a novel non-viral strategy for biotechnological as well as gene-therapy-related applications that require potent expression for extended time periods.

## Introduction

The transcription of protein-coding genes is a pivotal process underlying proper cellular function. The accurate initiation of transcription by RNA polymerase II (Pol II) is a critical step in the regulation of gene expression, in which Pol II is recruited to the core promoter via the basal transcription machinery (for a review, see [1, 2]). The core promoter is typically 80 nucleotides long, encompassing from -40 to +40 relative to the transcription start site. In the past, it was presumed that all core promoters function via a single universal mechanism, however, it is now well established that core promoters differ in both structure and function [3–13]. The core promoter consists of several functional subregions, termed core elements or motifs. Some of the known core promoter elements are the TATA box, TFIIB recognition elements (BREu and BREd), DCE, initiator (Inr), TCT, motif ten element (MTE) and DPE [14–26]. The TATA box, which is recognized and bound by the TBP subunit of the TFIID complex, is the best-known element [14]. The Inr motif is probably the most common element, and it encompasses the transcription start site (the A nucleotide in the Inr consensus is usually designated as position “+1”, whether or not the predominant site of initiation is at this nucleotide) [9, 20]. Both the MTE and DPE motifs are located downstream of the Inr and serve as recognition sites for the TAF6 and TAF9 subunits of TFIID [23, 25]. The MTE and DPE function in a cooperative manner with the Inr, and a precise spacing between the Inr and each of these motifs is crucial for transcriptional activity [22–25]. There is no universal core promoter composition, and different combinations of motifs confer specific functional properties to the core promoter, *e*.*g*. the ability to function in concert with specific enhancers [27–31] or regulate developmental gene regulatory networks [32, 33]. Moreover, there are core promoters that lack any of the known core motifs, suggesting the existence of additional core promoter elements that remain to be discovered.

Previous studies of the MTE motif revealed that although the MTE can function independently of the TATA box and DPE motifs, its collaboration with the TATA box, as well as with the DPE, results in strong synergy [22]. This synergy raised the idea that by combining different core promoter elements into a single core promoter, it could be possible to design unusually strong synthetic core promoters. We have previously designed synthetic core promoters, designated Super Core Promoters (SCP1 and SCP2), which contain the TATA box, Inr, MTE and DPE elements that drive high levels of transcription both *in-vivo* and *in-vitro* [34]; reviewed in [35]. To date, no natural promoters that contain such a combination of core promoter elements in a single promoter have been identified.

We now constructed an improved and potent SCP, termed SCP3. Chloramphenicol acetyltransferase (CAT) and luciferase reporter assays were used to assay the transcriptional activity of the abovementioned synthetic core promoters in the past [34]. Notably, unlike these methods that require harvesting of the cells at a specific time point, here we constructed *EGFP* expression vectors driven by distinct core promoters, including the previously characterized SCP1 and SCP2, and the novel SCP3. By the use of the *EGFP* reporter gene, it is possible to qualitatively follow the activity of the various core promoters in the same population of transfected living cells over time, and quantitatively analyze the activity of each promoter at any given time point.

In this study, we analyzed the expression driven by the various core promoters both by live cell imaging of continuously grown cells and in parallel, by flow cytometric analysis of cells harvested at specific time points, in three types of human cell lines for extended time periods. We demonstrate potent and prolonged gene expression using transient transfections (typically considered optimal 24–96 h following transfection). Thus, we provide a novel method for robust and long-term gene expression by the use of engineered super core promoters in transiently transfected mammalian cells.

## Materials and Methods

### Core promoter sequences and plasmids

*EGFP* expression plasmids containing the CMV enhancer were constructed using the pRc/CMV vector (Life Technologies), into which the *EGFP* reporter gene (Clonetech) was subcloned using restriction enzymes. The core promoter region of the commercial pRc/CMV, which contains the CMV TATA box and downstream vector sequences, was replaced with CMV, SCP2, or SCP3 sequences from –36 to +45 relative to the A+1 of the transcription start site. The pRc/CMV, natural CMV and SCP2 core promoters have been previously described [34]. Detailed information on the construction of all *EGFP* expression plasmids is provided in S1 Fig and S1 File. The sequences of pRc/CMV, natural CMV, SCP2 and the newly designed SCP3 are provided in Table 1. Notably, unlike the other three vectors, the SCP3 vector does not contain a T7 promoter between the core promoter and the *EGFP* reporter gene.

**Table 1:** A comparison of the four core promoters DNA sequences.

	-36…………………………..+1…………………………………..+45
pRc/CMV	AGGTC**TATATAAG**CAGAGCTCTCTGGCTAACTAGAGAACCCACTGCTTAACTGGCTTATCGAAAT
natural CMV	AGGTC**TATATAAG**CAGAGCTCGTTTAGTGAACCG**TCAGAT**CGCCTGGAGACGCCATCCACGCTGTTTTGACCTCCATAGAA
SCP2	AGGTC**TATATAAG**CAGAGCTCGTTTAGTGAACCG**TCAGAT**CGCCTGGAGACGT**CGAGCCGAGTGGTTGT**GCCTCCATAGAA
SCP3	AGGTC**TATATAAG**CAGAGCTCGTTTAGTGAACCG**TCAG****TC**CGCCTGGAGACCT**CGAGCCGAGTGGT****C****GT**GCCTCCATAGAA
	…..TATA-box………………….Inr………………MTE…..DPE…………..
	-36…………………………..+1…………………………………..+45
pRc/CMV	AGGTC**TATATAAG**CAGAGCTCTCTGGCTAACTAGAGAACCCACTGCTTAACTGGCTTATCGAAAT
natural CMV	AGGTC**TATATAAG**CAGAGCTCGTTTAGTGAACCG**TCAGAT**CGCCTGGAGACGCCATCCACGCTGTTTTGACCTCCATAGAA
SCP2	AGGTC**TATATAAG**CAGAGCTCGTTTAGTGAACCG**TCAGAT**CGCCTGGAGACGT**CGAGCCGAGTGGTTGT**GCCTCCATAGAA
SCP3	AGGTC**TATATAAG**CAGAGCTCGTTTAGTGAACCG**TCAG****TC**CGCCTGGAGACCT**CGAGCCGAGTGGT****C****GT**GCCTCCATAGAA
	…..TATA-box………………….Inr………………MTE…..DPE…………..

The sequences of pRc/CMV, natural CMV, SCP2 and SCP3 core promoters from –36 to +45 relative to the A+1 of the transcription start site are provided. Core promoter elements are marked in bold. Single nucleotide changes in SCP3 (relative to SCP2) are underlined. Notably, in the pRc/CMV vector, the sequence downstream of the -16 is artificial and the +1 marks a putative start site based on the spacing from the TATA box. An Inr sequence is present in the CMV natural promoter adjacent to the transcription initiation site [39, 40]. As a point of reference, the A in the Inr consensus is commonly designated as the +1 position in the promoter, whether or not the predominant site of initiation is at this nucleotide [9].

### Cell culture and transfection

HeLa S3 and SH-SY5Y cells were cultured in DMEM/F12 supplemented with 10% FBS and grown at 37°C with 5% CO_2_. For flow cytometric analysis and live cell imaging, HeLa S3 or SH-SY5Y cells were plated in 24-well plates one day prior to transfection. Cells were transfected with the various promoter–*EGFP* constructs by using the TransFast reagent (Promega) according to the manufacturer’s instructions. HeLa S3 cells were transfected with 1μg and SH-SY5Y cells were transfected with 0.75μg of each of the *EGFP* constructs. HOP-92 cell culture and transfection conditions are provided in Supporting information—Methods.

### Live cell imaging

For live cell imaging, HeLa S3 and SH-SY5Y cells were plated in 24-well plates one day prior to transfection. Cells were transfected with the different promoter–*EGFP* constructs as described, and incubated for 4 or 8 days. Cells were cultured for the duration of the experiments and the same population of cells was imaged by time-lapse microscopy every 24 h, starting 24 h after transfection for days 1–4, and 98 h after transfection for days 4–8 (imaging of days 1–4 and 4–8 was performed in separate experiments, in order to prevent cell overcrowding). Images were acquired using the Zeiss Observer Z1 inverted microscope with a 5X ECPlan-Neofluar objective, equipped with a 37°C 5% CO_2_ incubation chamber. In order to image a whole well, images were acquired with Zeiss AxioVision software’s “tiles” function, allowing stitching images corresponding to a field of a complete well. Resulting images were processed using Adobe Photoshop software.

### Flow cytometry

Flow cytometric analyses for short- and long-term experiments were performed separately (1–4 and 4–8 days post-transfection, respectively, so that day 4 is common to both analyses), to prevent cell overcrowding. HeLa S3 cells were plated at 2–7 x 10^4^ cells per well, and SH-SY5Y cells were plated at 3–9 x 10^4^ cells per well (depending whether the cells were to be analyzed by flow cytometry in days 1–4 or 4–8) in 24-well plates one day prior to transfection. Cells were transfected as described and incubated for 1–8 days. At the indicated time points, cells were harvested, centrifuged at 1000 rpm, resuspended in 0.3 mL PBS and subjected to flow cytometric analysis (Gallios, Beckman Coulter). The fluorescence of *EGFP* was measured in 10,000 cells per sample. Importantly, the flow cytometric analysis was performed using the same protocol, settings and conditions in every single experiment. The data was analyzed using the FlowJo software.

### Real-Time quantitative PCR

Real-Time quantitative PCR (qPCR) experiments were performed on days 2,4,6,8 and 14 post-transfection. HeLa S3 and SH-SY5Y cells were plated at 2–7 x 10^4^ and 3–9 x 10^4^ cells per well, respectively, in 24-well plates one day prior to transfection. Cells were transfected as described. At the specified time points, cells were harvested, centrifuged at 1000 rpm, washed in 0.4 mL PBS, centrifuged again at 500 rcf and frozen until further use. Total DNA used to validate the quality of the primers was purified using the ArchivePure DNA kit (5PRIME). Undiluted total DNA was subjected to qPCR. Plasmid DNA from transfected cells was purified using the TIANprep Rapid Mini Plasmid Kit (TIANGEN). Notably, while the purpose of this kit is to purify plasmid DNA from bacteria, we were able to use it and purify plasmid DNA from transfected mammalian cells. DNA preparations from different constructs at each time point yielded essentially the same DNA concentrations, enabling us to dilute all samples by the same factor. A total of 0.05ng DNA from each sample was subjected to qPCR analysis (StepOnePlus, Life Technologies) using SYBR Green (Life Technologies). The mean values of Ct (threshold cycle) for the GAPDH, *EGFP* and Neomycin genes were compared between each transfected construct, at the indicated time points.

Primer sequences for qPCR:

GAPDH Forward primer: CTATAAATTGAGCCCGCAGCCTCC, Reverse primer: CCCATGGTGTCTGAGCGATGTG

*EGFP* Forward primer: TCCAGGAGCGCACCATCTTC, Reverse primer: CGATGCCCTTCAGCTCGATGC

Neomycin Forward primer: CTTGCTCCTGCCGAGAAAGT, Reverse primer: GAGTACGTGCTCGCTCGATG.

### Statistical analysis

The fluorescence intensity and number of cells expressing *EGFP* for each promoter in each experiment was compared to the pRc/CMV data obtained in the corresponding experiment (by division of the obtained absolute value by that of the pRc/CMV promoter). Statistical comparisons between the promoters were done using the non-parametric Kruskal-Wallis test for independent samples, with pairwise comparisons. The Kruskal-Wallis test was chosen since it is a non-parametric test (which does not require the assumption that the samples are distributed normally) and since it can be applied to examine groups of unequal sizes. A p-value ≤ 0.05 was considered to be statistically significant.

## Results

### Generation of a new synthetic core promoter for enhanced gene expression

Synthetic core promoters, termed super core promoters (SCPs), which combine different promoter core elements into a single core promoter, have previously been shown to enable high levels of gene expression [34]. The highest transcriptional activity observed in these studies was achieved by using the SCP2 in concert with the CMV enhancer. In order to employ the SCP strategy to further enhance gene expression and to explore the temporal dynamics of SCPs’ function in conjunction with the CMV enhancer, we designed a new super core promoter, termed SCP3. Similarly to SCP2, SCP3 contains the CMV TATA and Inr motifs, along with the *D*.*melanogaster Tollo* MTE [22] and the human *Calm2* DPE [36]. Unlike SCP2, SCP3 does not contain the T7 promoter that is downstream of +45 relative to the +1 transcription start site to position the *EGFP* reporter gene immediately downstream of the promoter as the proximity might contribute to higher expression.

**Fig 1:**
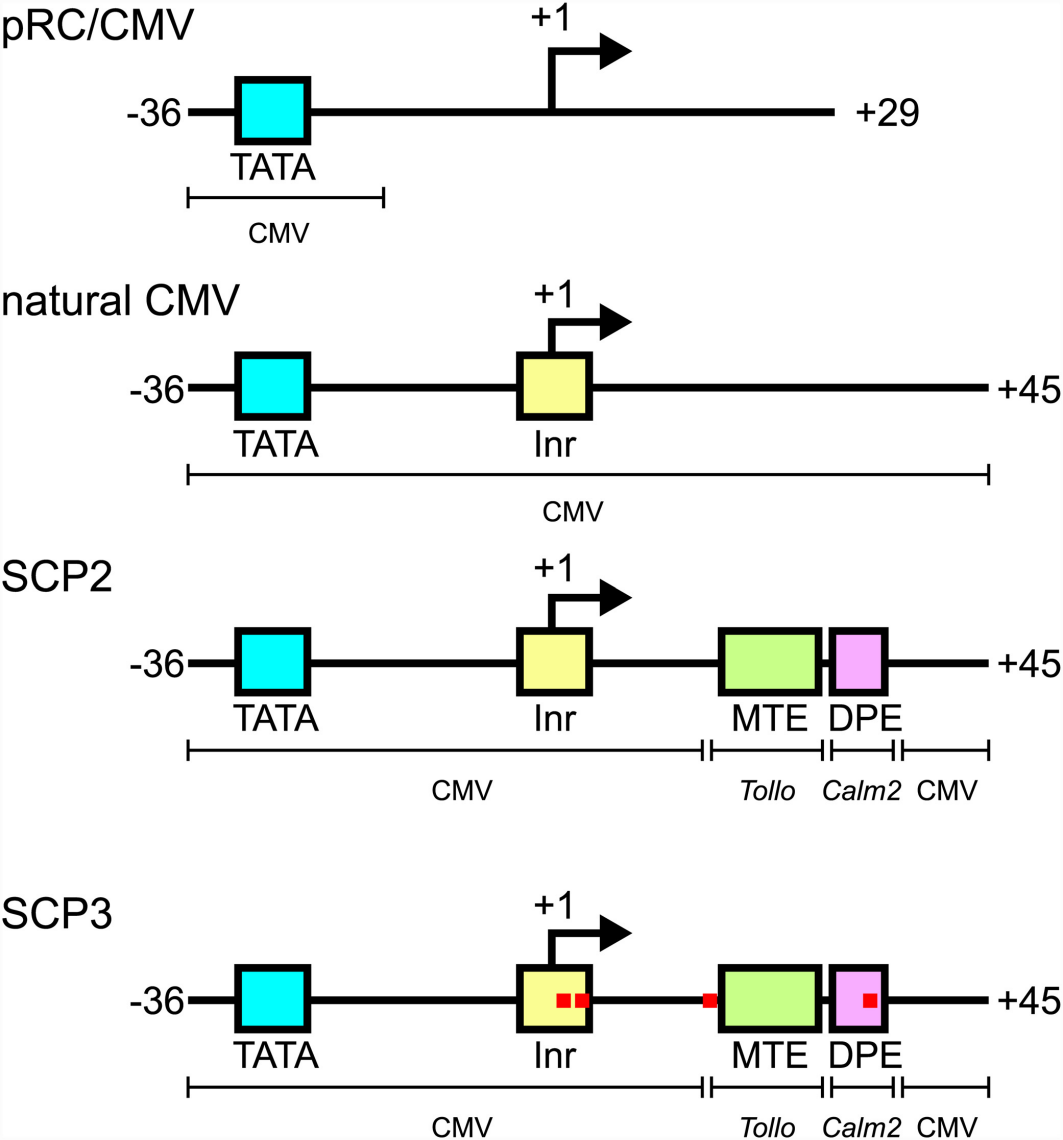
Schematic representation of the engineered core promoters. The pRc/CMV vector (Life Technologies) contains the CMV enhancer and TATA box, but lacks any CMV sequences that are downstream of -16 relative to the +1 transcription start site (including the Inr element). Three variants of pRc/CMV were constructed in which the core promoter region (from -36 to +45) was replaced with either the natural CMV core promoter, which contains the CMV TATA and Inr elements, or with SCP2 or SCP3, which contains the CMV TATA and Inr, the *Tollo* MTE, and the *Calm2* DPE. Single nucleotide changes in SCP3 (relative to SCP2) are marked by red rectangles. Each of these pRc/CMV-based constructs contains the *EGFP* reporter gene.

Additionally, SCP3 includes four nucleotide changes as compared with SCP2, as follows. To improve the Inr sequence two nucleotide changes were introduced: the A in position +3 was changed to T (as the T matches the *Drosophila* Inr consensus (TCA_+1_KTY) and is common in many DPE- and MTE- containing promoters), and the T in position +4 was changed to C (as it matches the Inr of both the human DPE-containing *Calm2* promoter [36] and the SCP1, which is stronger than SCP2 in the absence of an enhancer or in the presence of the SV40 enhancer [34]). To improve the sequence adjacent to the MTE, G in position +16 was changed to C (based on single nucleotide analysis of the *Tollo* MTE [23]). To improve the DPE, T in position +31 was changed to C (as many DPE- driven genes and several MTE-driven genes contain C in this position [22, 23, 26]) (Fig 1, Table 1). We designed and constructed four pRc/CMV-based expression vectors, each driving the expression of the same *EGFP* reporter gene, in order to test the effects of the different core promoters on transcriptional activity: the incomplete CMV (pRc/CMV), the complete CMV (natural CMV), the SCP2 [34], and the novel SCP3 (S1 Fig and Supporting information -Methods).

### Live cell imaging reveals strong *EGFP* expression under the regulation of SCP3

A distinct increase in transcriptional activity was previously demonstrated using engineered core promoters, favoring the natural CMV and SCP2 core promoters in conjunction with the CMV enhancer, relative to the pRc/CMV (Life Technologies) core promoter [34]. These studies utilized the chloramphenicol acetyltransferase (CAT) and luciferase reporter genes, and cells were assayed 24–48 h post-transfection for CAT and luciferase activities. Unlike these assays, which require harvesting of the cells at a specific time point, here we took advantage of the *EGFP* reporter gene that enables us to qualitatively follow the activity of the various core promoters in the same population of transfected living cells over time.

**Fig 2:**
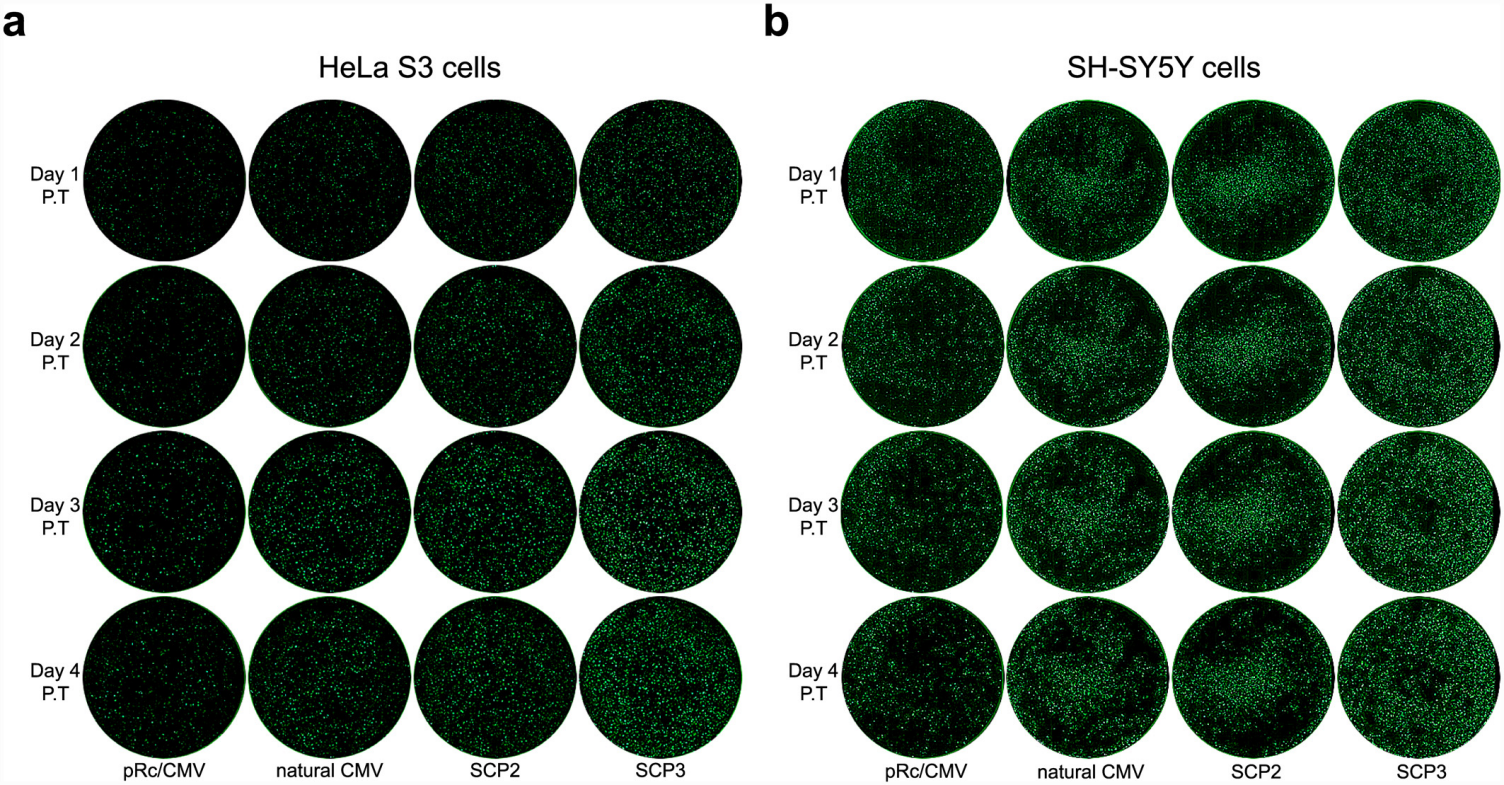
Live cell *EGFP* imaging of short-term expression of pRc/CMV-based constructs, in HeLa S3 and SH-SY5Y cells. HeLa S3 and SH-SY5Y cells were transiently transfected with either the pRc/CMV, natural CMV, SCP2 or SCP3 vector expressing *EGFP*. The cells were imaged once a day during 1–4 days post-transfection (P.T.). Each circle displays the whole well image constructed by stitching individual microscopic fields. (A) HeLa S3 cells. (B) SH-SY5Y cells. Data shown are representative of 3 independent experiments for each cell type.

To assess the transcription activity of the core promoters, we transiently transfected two types of human cell lines, HeLa S3 (human cervical carcinoma cells) and SH-SY5Y (human neuroblastoma cells originating from bone marrow tissue), with the pRc/CMV, natural CMV, SCP2 or SCP3-driven *EGFP* expression vectors, and imaged the fluorescence signals expressed by the transfected cell populations. It is of note that the CMV promoter is considered one of the strongest naturally occurring promoters, and it is frequently used for overexpression vectors in multiple settings. Live cell imaging for short-term expression, performed during days 1–4 post-transfection, reveals that full length core promoters have a substantial advantage over the commercial pRc/CMV core promoter, both in terms of fluorescence intensity and number of fluorescent cells (Fig 2). During the 4 days after transfection, an increase in the fluorescence levels, which is indicative of the transcriptional activity, can be observed in cells transfected with all four promoters, though only slightly in the pRc/CMV promoter. Interestingly, SCP3 is consistently the most active core promoter among the four.

**Fig 3:**
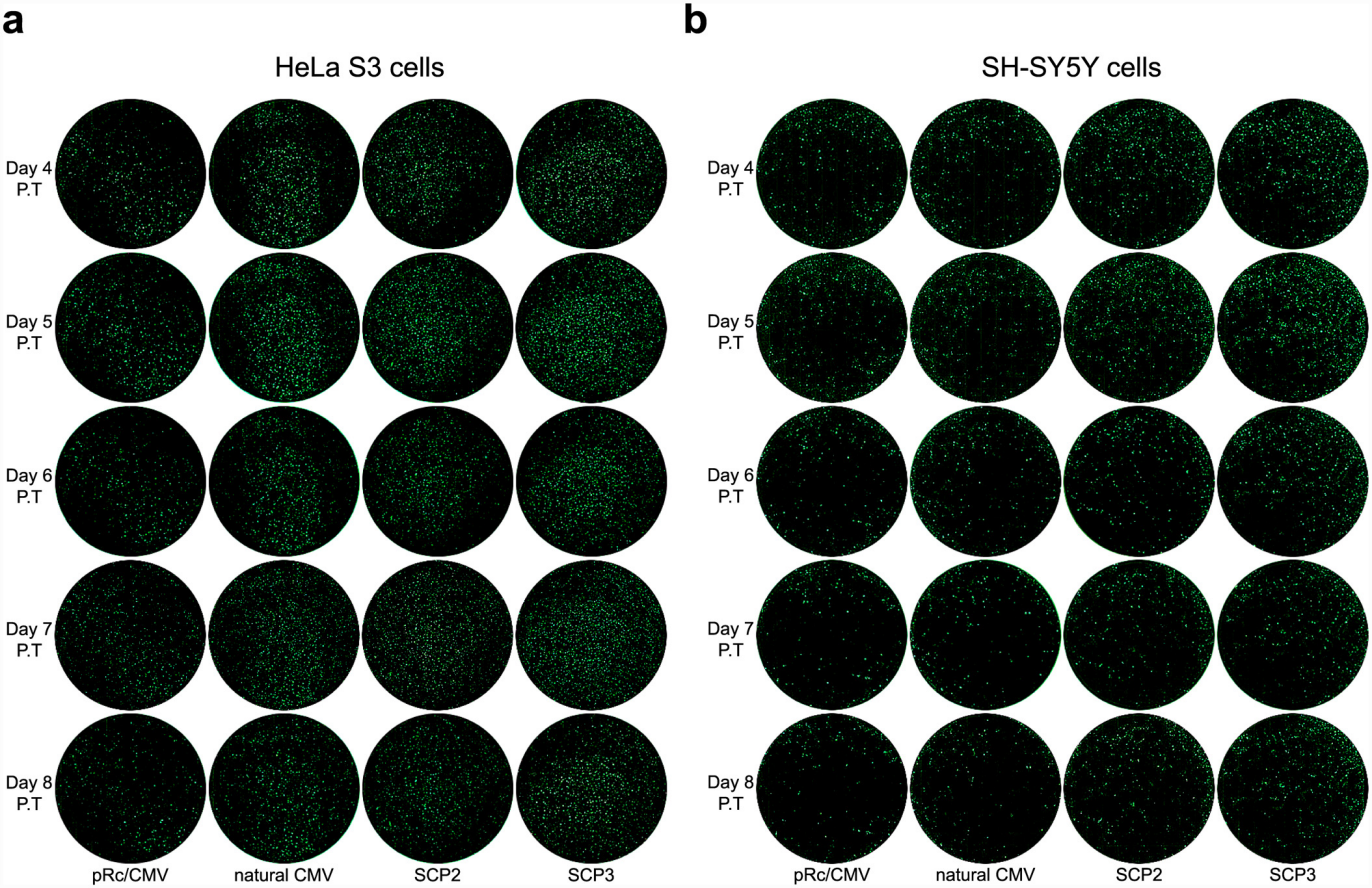
Live cell *EGFP* imaging of long-term expression of pRc/CMV-based constructs, in HeLa S3 and SH-SY5Y cells. HeLa S3 and SH-SY5Y cells were transiently transfected with either the pRc/CMV, natural CMV, SCP2 or SCP3 vector expressing *EGFP*. The cells were imaged once a day during 4–8 days post-transfection (P.T.). Each circle displays the whole well image constructed by stitching individual microscopic fields. (A) HeLa S3 cells. (B) SH-SY5Y cells. Data shown are representative of 4 independent experiments for each cell type.

To examine whether these promoters drive long-term expression, live cell imaging was performed during days 4–8 post-transfection. Remarkably, a distinct increase in activity was observed at these later time points using the complete natural CMV, SCP2 and especially SCP3, as compared to the commercial pRc/CMV core promoter (Fig 3). As expected of transient transfections, we observed a decrease in fluorescence intensity during the long term. However, in days 7 and 8, there are still relatively high fluorescence intensities driven by the full length core promoters, and particularly the SCP3 core promoter. Remarkably, transiently transfected cells express *EGFP* for longer time periods, as some SCP3-driven *EGFP* SH-SY5Y cells remain fluorescent even 4 weeks post-transfection (S2 Fig).

### Quantitative flow cytometric analysis reveals strong *EGFP* expression under the regulation of SCP3

**Fig 4:**
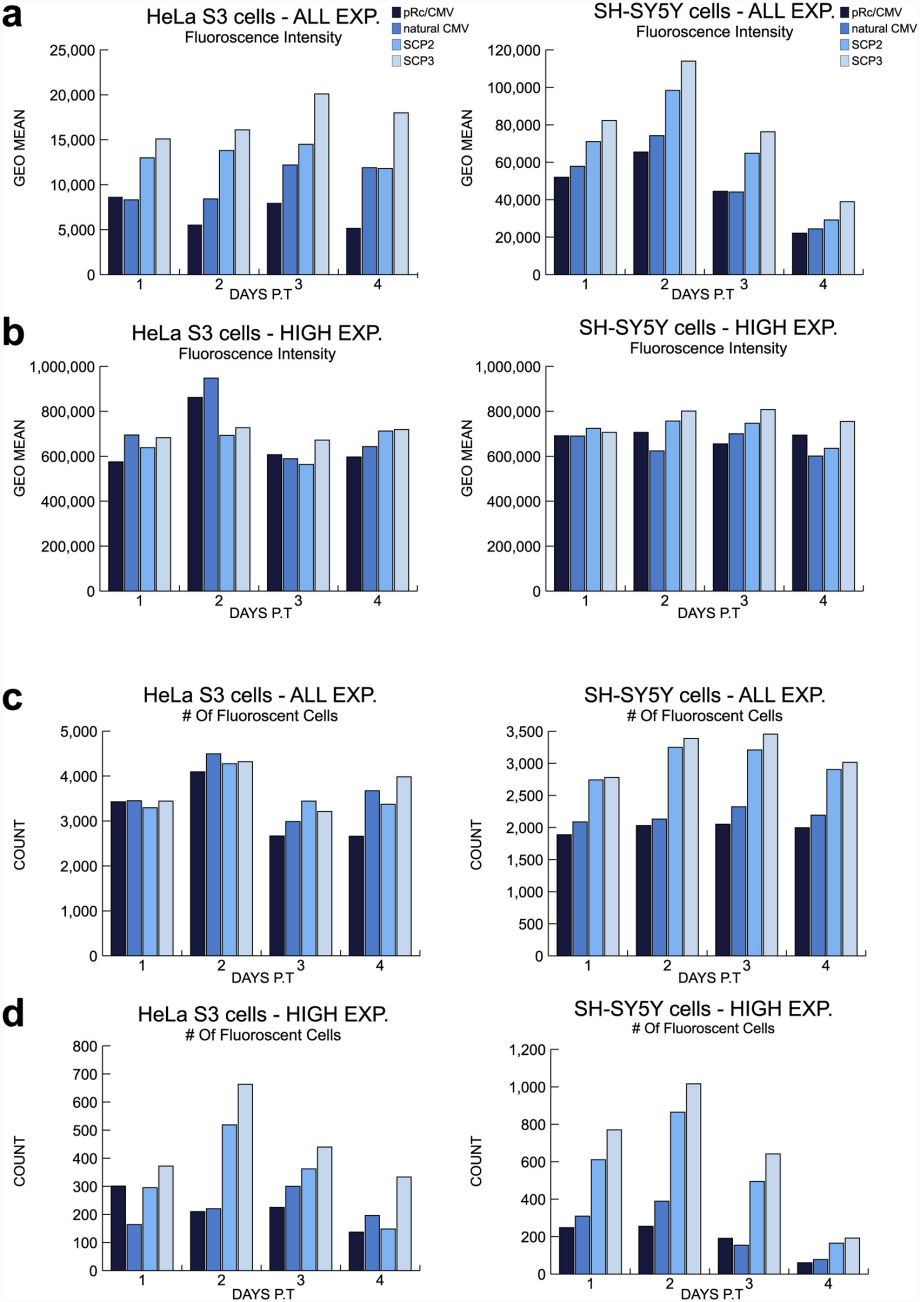
Flow cytometric analysis of short-term fluorescence intensity and number of fluorescent HeLa S3 and SH-SY5Y cells. HeLa S3 and SH-SY5Y cells were transiently transfected with pRc/CMV, natural CMV, SCP2 or SCP3 vector expressing *EGFP*. The cells were collected 1–4 days post-transfection (P.T.) for flow cytometric analysis. (A) Flow cytometric analysis of fluorescence intensity of all HeLa S3 fluorescent cells and SH-SY5Y fluorescent cells. (B) Flow cytometric analysis of fluorescence intensity of high intensity HeLa S3 fluorescent cells and SH-SY5Y fluorescent cells. (C) Flow cytometric analysis of the number of all HeLa S3 fluorescent cells and SH-SY5Y fluorescent cells. (D) Flow cytometric analysis of the number of high intensity HeLa S3 fluorescent cells and fluorescent SH-SY5Y cells. Data shown are representative of 5 independent experiments using HeLa S3 cells and 6 independent experiments using SH-SY5Y cells. Statistical comparisons between the promoters were done using the Kruskal—Wallis test with pairwise comparisons. Significant p-values (p ≤0.05) are indicated in the results section.

**Fig 5:**
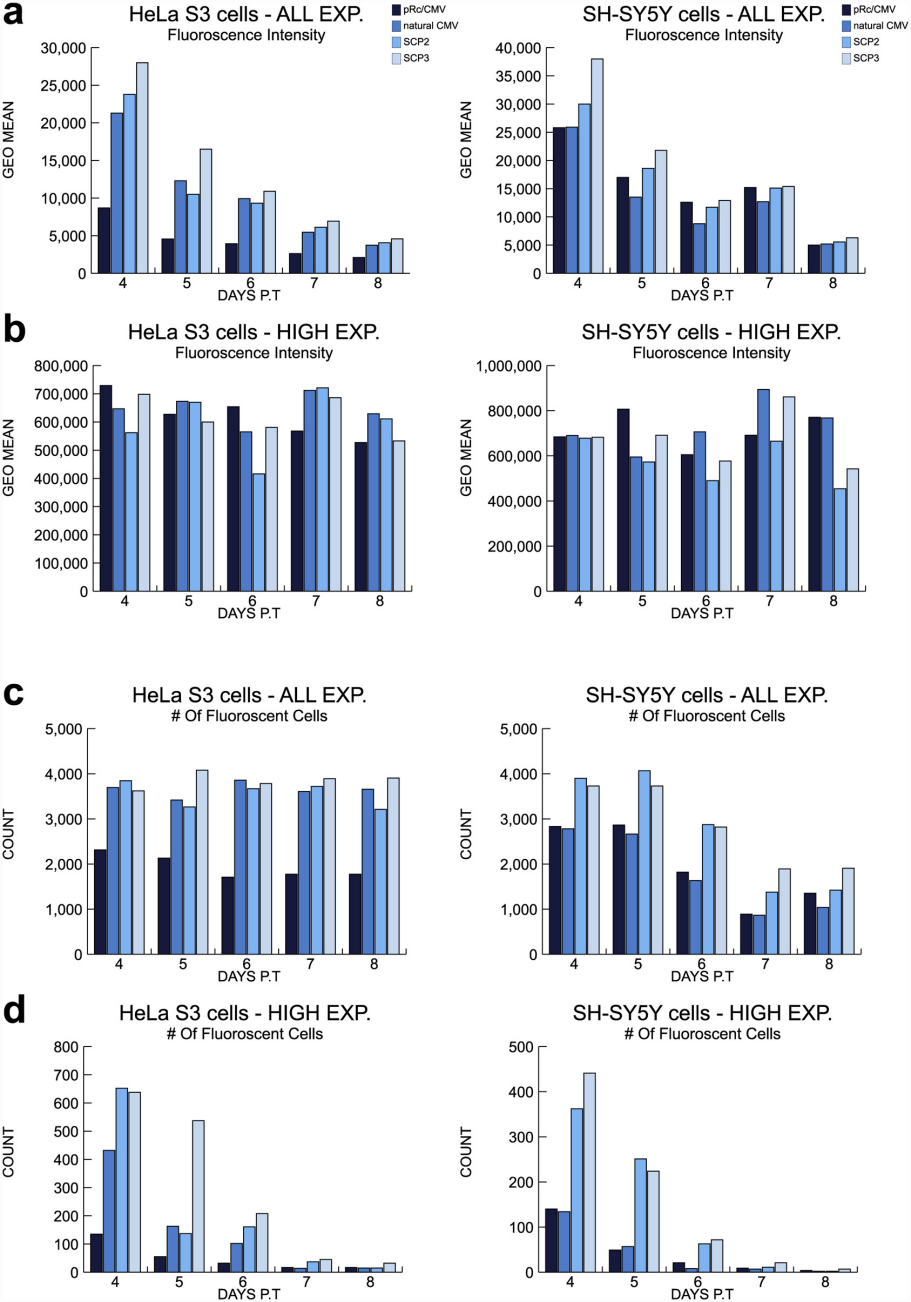
Flow cytometric analysis of long-term fluorescence intensity and number of fluorescent HeLa S3 and SH-SY5Y cells. HeLa S3 and SH-SY5Y cells were transiently transfected with pRc/CMV, natural CMV, SCP2 or SCP3 vector expressing *EGFP*. The cells were collected 4–8 days post-transfection (P.T.) for flow cytometric analysis. (A) Flow cytometric analysis of fluorescence intensity of all HeLa S3 fluorescent cells and SH-SY5Y fluorescent cells. (B) Flow cytometric analysis of fluorescence intensity of high intensity HeLa S3 fluorescent cells and SH-SY5Y fluorescent cells. (C) Flow cytometric analysis of the number of all HeLa S3 fluorescent cells and SH-SY5Y fluorescent cells. (D) Flow cytometric analysis of the number of high intensity HeLa S3 fluorescent cells and SH-SY5Y fluorescent cells. Data shown are representative of 6 independent experiments using HeLa S3 cells and 5 independent experiments using SH-SY5Y cells. Statistical comparisons between the promoters were done using the Kruskal—Wallis test with pairwise comparisons. Significant p-values (p ≤0.05) are indicated in the results section.

To investigate the transcription activity of the various core promoters in a quantitative manner, we performed flow cytometric analysis. HeLa S3 and SH-SY5Y cells were transiently transfected with the pRc/CMV, natural CMV, SCP2 or SCP3-driven *EGFP* -expression vectors, and analyzed by flow cytometry on a daily basis for 8 days post-transfection (divided into short-term (d1-4) and long-term (d4-8) follow-up). To quantitatively measure the reporter levels of transcription activity and the number of cells expressing the reporter under the regulation of the four core promoters, we examined both the fluorescence intensity and the number of cells expressing *EGFP*. A non-parametric Kruskal-Wallis test was performed to examine the statistical differences between any pair of promoters. As the fluorescence intensity and the number of *EGFP*-expressing cells differ between cells expressing high fluorescence (high expressors) and low fluorescence expressors, we analyzed the cells that express high fluorescence levels (designated “HIGH EXP”) in addition to analyzing the entire population of fluorescent cells (designated “ALL EXP”). The flow cytometric analyses depicted are of one representative experiment for each short-term- (Fig 4) and long-term-set of experiments (Fig 5) out of 5–6 independent individual experiments for each cell line in each time frame. The average fold difference (±SEM) of the reporter gene expression driven by each promoter, as compared to the expression driven by the commercial pRc/CMV vector, is depicted in S3 Fig (short-term expression) and S4 Fig (long-term expression). The statistical significance was calculated for 5–6 individual experiments for each cell line for short- as well as long-term expression.

Flow cytometric analysis of short-term *EGFP*-expression reveals a constant advantage in favor of the SCP3 promoter (Fig 4 and S3 Fig). This advantage is reflected in both HeLa S3 and SH-SY5Y cell lines, by the fluorescence intensity levels of all fluorescent cells. Analysis of the fluorescence intensity of all HeLa S3 fluorescent cells reveals that SCP3 is significantly stronger than the pRc/CMV promoter during all four days (n = 5, p ≤ 0.05). Similarly, analysis of all *EGFP*-expressing SH-SY5Y cells reveals that SCP3 is significantly stronger than pRc/CMV in days 1 and 2 post-transfection (n = 6, p ≤ 0.05) (Fig 4A and S3A Fig). Overall, we did not observe significant difference in the fluorescence intensity driven by either promoter when we analyzed the high expressors in both cell lines (Fig 4B and S3B Fig). This can be explained by the fact that this analysis only takes into account the cells that express very high levels, which are only a minority of the cells, as can be seen by comparing the number of the high fluorescence expressing cells (Fig 4D) to the total number of fluorescent cells (Fig 4C).

The advantage of SCP3 is also reflected in the number of all *EGFP*-expressing SH-SY5Y cells, where significant differences between SCP3 and pRc/CMV were observed during days 1, 2 and 4 post-transfection (n = 6, p ≤ 0.05) (Fig 4C and S3C Fig). The analysis of the number of high fluorescence intensity HeLa S3 and SH-SY5Y cells reveals significant advantage favoring SCP3 over the pRc/CMV promoter, in all four examined days (HeLa S3: n = 5, SH-SY5Y: n = 6, p ≤ 0.05) (Fig 4D and S3D Fig).

In addition to the advantage of SCP3, short-term analysis also reveals a significant advantage in favor of SCP2-driven transcription compared to the pRc/CMV promoter. This advantage is observed by the fluorescence intensity levels of all fluorescent HeLa S3 cells during days 2 and 4 (n = 5, p ≤ 0.05) (Fig 4A and S3A Fig). In terms of the number of *EGFP-*expressing HeLa S3 cells, we observed temporal variations upon comparison of SCP2 to pRc/CMV, however the number of all *EGFP-*expressing SH-SY5Y cells driven by SCP2 is significantly higher than pRc/CMV in days 2 and 4 (n = 6, p ≤ 0.05) (Fig 4C and 4D and S3C Fig).

Remarkably, flow cytometric analysis of long-term expression (performed during days 4–8 post-transfection) reveals an advantage of SCP3 over the pRc/CMV promoter in both HeLa S3 and SH-SY5Y cells. SCP3 exhibits a significant advantage over the pRc/CMV promoter in the entire population of fluorescent HeLa S3 cells in days 4–7 (n = 6, p ≤ 0.05) (Fig 5A and S4A Fig). The fluorescence intensity of all *EGFP*-expressing SH-SY5Y cells also reveals stronger transcriptional activity of the SCP3 promoter as compared to pRc/CMV, in days 4, 5 and 8 (n = 5, p ≤ 0.05) (Fig 5A and S4A Fig). Analysis of the fluorescence intensity of HeLa S3 and SH-SY5Y cells that express high levels of *EGFP* does not indicate constant significant differences among the promoters (Fig 5B and S4B Fig). As explained above, this results from the fact that the high expressors are only a minority of the cells, as can be seen by comparing the number of the high fluorescence expressing cells (Fig 5D) to the total number of fluorescent cells (Fig 5C).

SCP3 directs the long-term expression of a larger number of HeLa S3 and SH-SY5Ycells that express *EGFP*, as compared to the pRc/CMV promoter (Fig 5C and 5D, S4C and S4D Fig). This potent activity of SCP3 as compared to pRc/CMV in terms of a larger number of *EGFP*-expressing cells, is observed in days 5–8 of all fluorescent HeLa S3 cells and in days 4, 6 and 8 of all fluorescent SH-SY5Y cells (HeLa S3: n = 6, SH-SY5Y: n = 5, p ≤ 0.05) (Fig 5C and S4C Fig). The advantage of SCP3 over pRc/CMV in terms of expression in a larger number of cells, is also observed during days 4, 5 and 7 of the high *EGFP*-expressing HeLa S3 cells, and days 4, 5, 6 and 8 of the high *EGFP*-expressing SH-SY5Y cells (HeLa S3: n = 6, SH-SY5Y: n = 5, p ≤ 0.05) (Fig 5D and S4D Fig).

SCP2-driven transcription is significantly stronger as compared to the pRc/CMV promoter in terms of fluorescence intensity levels of all *EGFP*-expressing HeLa S3 cells during days 4, 6 and 7 (n = 6, p ≤ 0.05) (Fig 5A and S4A Fig). The advantage of SCP2 over pRc/CMV is also reflected by the number of HeLa S3 *EGFP*-expressing cells in days 5, 7 and 8 (n = 6, p ≤ 0.05) (Fig 5C and S4C Fig).

Notably, we have performed similar flow cytometric experiments to assay the expression driven by these promoter variants in a third human cell line (HOP-92; non-small cell lung carcinoma). As can be seen in S5 Fig, SCP3 is very potent as compared to the pRc/CMV both in terms of fluorescence intensity levels and the number of *EGFP*-expressing cells. In addition, SCP3 is overall stronger in these cells than the natural CMV and SCP2, whose activities are higher (albeit, not statistically significant) than the pRc/CMV.

**Fig 6:**
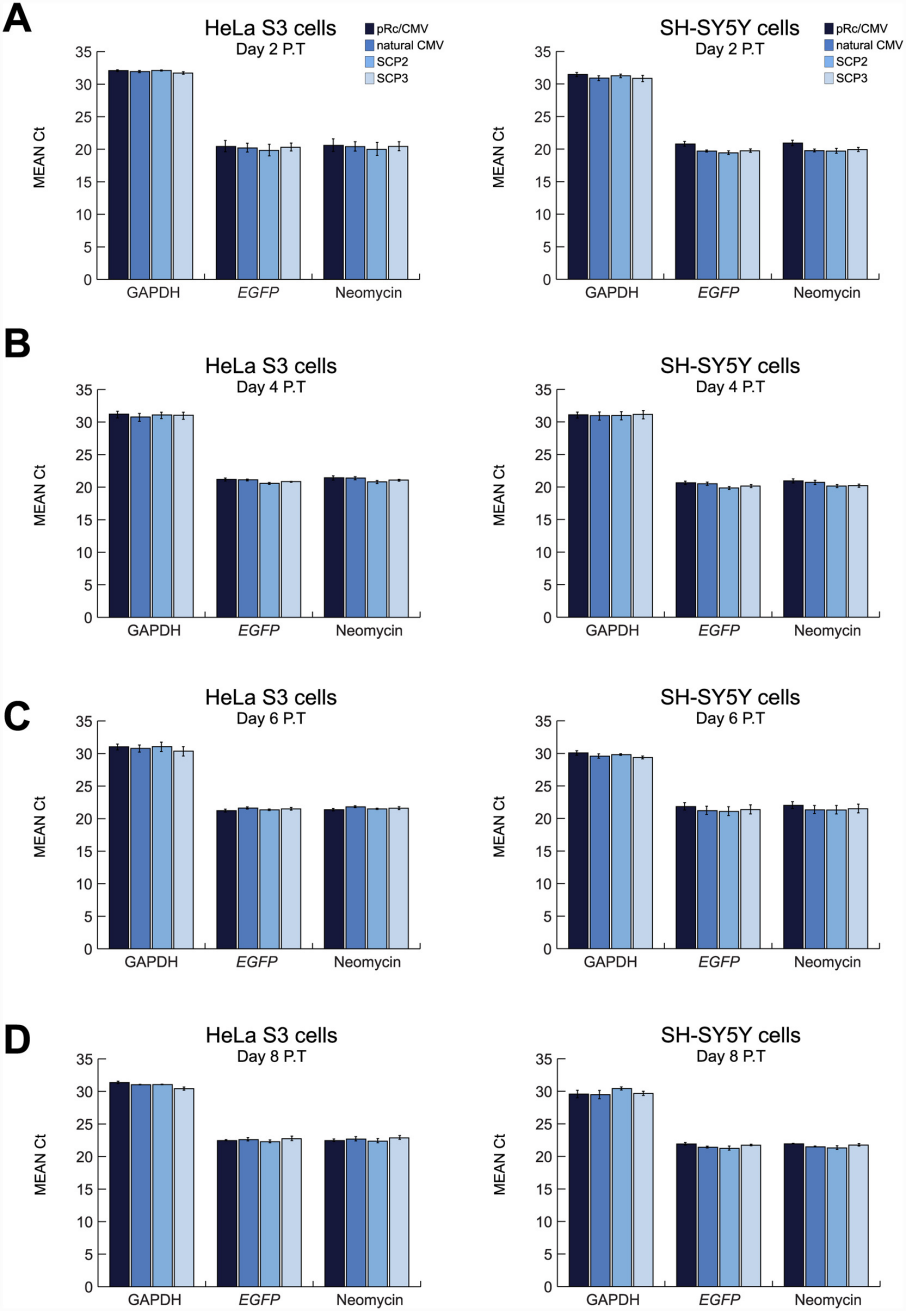
Real-Time quantitative PCR of purified transiently transfected plasmid DNA in HeLa S3 and SH-SY5Y cells. HeLa S3 and SH-SY5Y cells were transiently transfected with pRc/CMV, natural CMV, SCP2 or SCP3 vector expressing *EGFP*, and harvested every other day during 8 days post-transfection (P.T.). Plasmid DNA was purified from cells and subjected to qPCR analysis with primers for the GAPDH, *EGFP* and Neomycin genes. Data shown are the averaged Ct values of 3 independent experiments (each performed in triplicates). (A) 2 days post-transfection. (B) 4 days post-transfection. (C) 6 days post-transfection. (D) 8 days post-transfection. Error bars represent SEM.

In order to verify that the measured *EGFP* signals are indeed a function of the transcription activity of the various promoters as opposed to differences in DNA uptake, we performed Real-Time quantitative PCR (qPCR) assays using plasmid DNA purified from transfected HeLa S3 and SH-SY5Y cells as templates after 2, 4, 6 or 8 days following transfection. We used primers for the endogenous GAPDH gene, as well as primers for the *EGFP* and Neomycin genes originating from the transfected vectors. The threshold cycle (Ct) values for each target gene were compared in order to assess the levels of genomic and plasmid DNA in the samples. Importantly, all transfections were done under similar conditions using the same amounts of DNA, thus variations in DNA uptake among the various constructs would result in different Ct values. The data represents the average of three independent qPCR experiments (each carried out in triplicates) for each cell line in each time point. The levels of GAPDH were analyzed as a measure of the presence of genomic DNA in the samples. The Ct values using GAPDH primers (~29.5–32.0) were similar to values obtained with no DNA template controls, indicating the successful isolation of plasmid DNA from the transfected mammalian cells (Fig 6 and S1 Table). Notably, these GAPDH primers are capable of detecting the GAPDH gene in a total DNA preparation (Ct values of 24–25; S6 Fig). The Ct values obtained from all samples using primers for both *EGFP* and Neomycin ranged from 19.5 to 22, indicating the amplification of plasmid DNA rather than genomic DNA (Fig 6 and S1 Table). Importantly, the Ct values obtained using both *EGFP* and Neomycin primers are comparable among all constructs in each time point (Fig 6). Furthermore, the Ct values obtained for *EGFP* are comparable to those of Neomycin per each construct and time point, indicating that each purified plasmid contains both the *EGFP* gene and the Neomycin gene, as expected.

**Fig 7:**
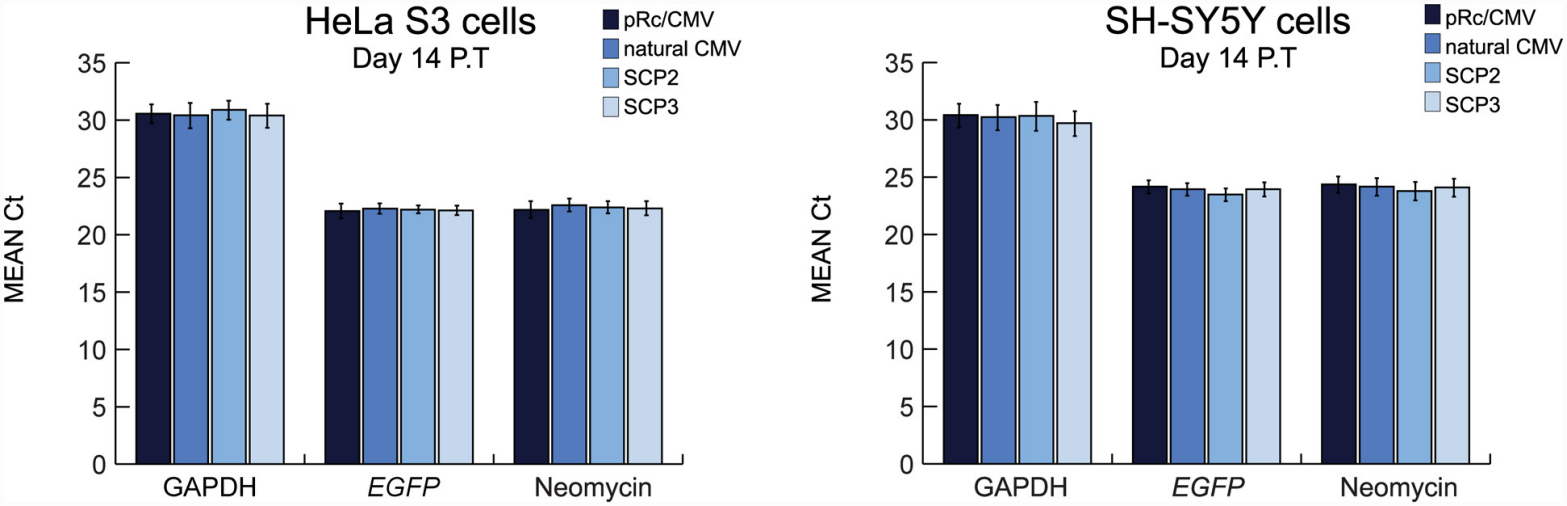
Real-Time quantitative PCR of transiently transfected plasmid DNA purified 2 weeks post-transfection of HeLa S3 and SH-SY5Y cells. HeLa S3 and SH-SY5Y cells were transiently transfected with pRc/CMV, natural CMV, SCP2 or SCP3 vector expressing *EGFP*, and harvested 14 days post-transfection (P.T.). Plasmid DNA was purified from cells and subjected to qPCR analysis with primers for the GAPDH, *EGFP* and Neomycin genes. Data shown are the averaged Ct values of 3 independent experiments (each performed in triplicates). Error bars represent SEM.

Additionally, as some transiently transfected cells express *EGFP* for remarkably long periods of time (S2 Fig), we wanted to examine whether transfected plasmid DNA remains intact beyond 8 days post-transfection. To address this possibility, we implemented a similar qPCR approach for cells harvested 14 days post-transfection. Consistent with the Ct values obtained for the first 8 days (Fig 6), the Ct values at 14 days post-transfection for the GAPDH gene were ~30–31, while the Ct values of the *EGFP* and Neomycin genes ranged from 22 to 24 (Fig 7 and S1 Table). These findings demonstrate the successful purification of intact plasmid DNA. Overall, the similarities in Ct values obtained for the various promoters and the fact that plasmid DNA can still be obtained from cells harvested 14 days post-transfection, indicate that the observed prolonged and divergent *EGFP* expression levels do not result from differences in DNA uptake.

Taken together, the observed differences in *EGFP* expression driven by the various constructs result from differences in their transcriptional activity. SCP3 is superior to the pRc/CMV. Notably, it is also stronger than the natural CMV and SCP2, and its advantage over these promoters is cell line-dependent. Importantly, using the newly engineered synthetic promoter SCP3, and in some cases also SCP2, we achieved robust and long-term *EGFP* expression, as reflected by the fluorescence intensity and the overall number of *EGFP* expressing cells, in transiently transfected HeLa S3, SH-SY5Y and HOP-92 cells.

## Discussion

The use of SCPs, which combine different core promoter elements into a single core promoter, demonstrate that the core promoter is a regulatory module that can not only affect the initiation of transcription, but also achieve high levels of transcription [34]. In this study, we investigated and compared the transcription activity of four core promoters: the pRc/CMV, the natural CMV, SCP2 and the newly engineered SCP3, and their abilities to drive enhanced long-term gene expression in three different transiently transfected mammalian cell lines.

Qualitative analysis (using live cell imaging) and quantitative analysis (using flow cytometry) revealed a distinct increase in transcriptional activity in favor of core promoters containing both upstream and downstream elements, particularly the SCP3. This advantage is reflected both by high expression levels and by the high number of cells that express *EGFP*. In addition, by the use of the *EGFP* reporter gene, we were able to add a new, temporal dimension to the characterization of gene expression driven by the SCPs.

There are several indications that integration of plasmid DNA into chromosomal DNA is unlikely to account for the prolonged expression driven by the various constructs. First, cells were transiently transfected and no antibiotic selection was used. Second, the fluorescence levels of all *EGFP* expressing cells substantially decline after 6 days. Importantly, qPCR analysis of DNA purified from transfected cells throughout the experiment and even at 14 days post-transfection, revealed the existence of substantial amounts of intact plasmid DNA in the cells.

Importantly, we discovered that under the regulation of the full-length core promoters and particularly SCP3, gene expression is considerably prolonged. Thus, the SCPs provide a novel non-viral way for long-term gene expression in transiently transfected cells. This can be relevant in cases where long-term follow-up is important, such as the response to drugs and cytokines.

A modified core promoter has previously been employed in *Drosophila* to identify different enhancers and the expression patterns they drive in small subsets of neurons, for manipulation and dissection of neural circuitry [37]. As enhancers may require specific core promoter elements in order to function properly, it was instrumental to generate transgenic *Drosophila* lines by combining genomic fragments that contain putative enhancers, which are known to have brain-specific expression patterns, with a synthetic *Drosophila* SCP-like promoter (DSCP) that is designed to work with a wide range of enhancers. This DSCP is an example to the versatility of designing new SCPs that can function with more than one enhancer. Hence, new SCPs could be designed, both in conjunction with enhancers that can either provide a more specific or alternatively, permissive expression.

Interestingly, another study that utilized the SCP strategy to perform cryo electron microscopy, discovered that the human basal transcription factor TFIID coexists in two distinct structural conformations, the well-known canonical conformation and a novel rearranged conformation, where the rearranged state is important for the assembly of the preinitiation complex [38]. This structural transition is stimulated by the presence of TFIIA that facilitates the binding of TFIID to the core promoter, which occurs by independently interacting with different combinations of the TATA box, Inr, MTE and DPE core promoter motifs. The authors have shown that the core promoter architecture dictates TFIIA-dependent and -independent interactions of TFIID with core promoter DNA. Our results of high level and long-term transcription driven by the SCPs provide additional support for the central role of the core promoter composition in the regulation of gene expression and transcription initiation.

As SCP3 differs from SCP2 in the absence of the T7 promoter as well as in substitutions of four nucleotides while the composition of core promoter elements is the same, the high transcriptional activity observed for SCP3-driven *EGFP* indicates that the entire core promoter sequence, and not just the presence of the core promoter elements themselves, is important for core promoter function. However, since the T7 promoter is absent in the SCP3-driven construct, we cannot rule out the possibility that the high transcriptional activity of SCP3 may result, at least in part, from the proximity of the core promoter to the *EGFP* reporter gene. Regardless of the proximity (between the reporter gene and the promoter) effect, optimization of existing SCPs could be utilized to further enhance gene expression. We have successfully used the SCPs in three different types of human cell lines and obtained varying *EGFP* expression levels in these cell lines indicating that, as expected, the cell lines used may influence gene expression levels. Yet, expression of genes driven by SCP3 is superior to expression driven by previously tested core promoters. Importantly, the engineering of core promoter sequences to drive robust and long-term gene expression in transiently transfected cells provides a novel means for biotechnological and gene-therapy-related applications.

## Supporting Information

S1 Fig Schematic illustration of the constructed *EGFP* expression plasmids driven by the various core promoters linked to the CMV enhancer. The *EGFP* gene was cloned into the HindIII and XbaI sites of the commercial pRc/CMV plasmid (Life Technologies). The CMV enhancer was amplified using PCR and the promoters of the natural CMV, SCP2 and SCP3 were cloned into the vector by “dropping in” annealed oligonucleotides and preserving the natural context of the natural CMV enhancer and promoter (*i*.*e*., no restriction sites or any artificial spacers were introduced between the enhancer and the promoters). Sequences derived from CMV are shaded in gray; artificial pRc/CMV vector sequence is colored white; MTE and DPE sequences are shaded in yellow. Red lines represent nucleotide changes from SCP2 to SCP3.
(PDF)

S2 Fig Live cell imaging of SH-SY5Y cells expressing *EGFP* that is driven by pRc/CMV-based constructs for over 30 days. SH-SY5Y cells were transiently transfected with either pRc/CMV, natural CMV, SCP2 or SCP3 vector expressing *EGFP*. The cells were imaged over a 31 days period post-transfection (P.T.). Each circle displays the whole well image constructed by stitching individual microscopic fields. Data shown are representative of 2 independent experiments.
(PDF)

S3 Fig Flow cytometric analysis of short-term average fluorescence intensity and average number of fluorescent HeLa S3 and SH-SY5Y cells. HeLa S3 and SH-SY5Y cells were transiently transfected with pRc/CMV, natural CMV, SCP2 or SCP3 vector expressing *EGFP*. The cells were collected 1–4 days post-transfection (P.T.) for flow cytometric analysis. (A) Flow cytometric analysis of average fluorescence intensity of all HeLa S3 fluorescent cells and SH-SY5Y fluorescent cells. (B) Flow cytometric analysis of average fluorescence intensity of high intensity HeLa S3 fluorescent cells and SH-SY5Y fluorescent cells. (C) Flow cytometric analysis of the average number of all HeLa S3 fluorescent cells and SH-SY5Y fluorescent cells. (D) Flow cytometric analysis of the average number of high intensity HeLa S3 fluorescent cells and fluorescent SH-SY5Y cells. For each day, the measurements were normalized to the value measured for the pRc/CMV expressing *EGFP* vector at the corresponding day. Data shown are the average of 5 independent normalized experiments using HeLa S3 cells, and 6 independent normalized experiments using SH-SY5Y cells. Error bars represent SEM. Statistical comparisons between the promoters were done using the Kruskal—Wallis test with pairwise comparisons. * p ≤0.05, ▲ p ≤0.01(Black- compared to pRc/CMV, Green- compared to natural CMV).
(PDF)

S4 Fig Flow cytometric analysis of long-term average fluorescence intensity and average number of fluorescent HeLa S3 and SH-SY5Y cells. HeLa S3 and SH-SY5Y cells were transiently transfected with pRc/CMV, natural CMV, SCP2 or SCP3 vector expressing *EGFP*. The cells were collected 4–8 days post-transfection (P.T.) for flow cytometric analysis. (A) Flow cytometric analysis of average fluorescence intensity of all HeLa S3 fluorescent cells and SH-SY5Y fluorescent cells. (B) Flow cytometric analysis of average fluorescence intensity of high intensity HeLa S3 fluorescent cells and SH-SY5Y fluorescent cells. (C) Flow cytometric analysis of the average number of all HeLa S3 fluorescent cells and SH-SY5Y fluorescent cells. (D) Flow cytometric analysis of the average number of high intensity HeLa S3 fluorescent cells and fluorescent SH-SY5Y cells. For each day the measurements were normalized to the value measured for the pRc/CMV expressing *EGFP* vector at the corresponding day. Data shown are the average of 6 independent normalized experiments of all *EGFP*-expressing cells and 3 independent normalized experiments of high *EGFP*-expressing cells using HeLa S3 cells (see supporting methods for an explanation) and of 5 independent normalized experiments using SH-SY5Y cells. Error bars represent SEM. Statistical comparisons between the promoters were done using the Kruskal—Wallis test with pairwise comparisons. * p ≤0.05, ▲ p ≤0.01(compared to pRc/CMV).
(PDF)

S5 Fig Flow cytometric analysis of short and long -term average fluorescence intensity and average number of fluorescent HOP-92 cells. HOP-92 cells were transiently transfected with pRc/CMV, natural CMV, SCP2 or SCP3 vector expressing *EGFP*. The cells were collected 1–4 and 4–8 days post-transfection (P.T.) for flow cytometric analysis. (A) Flow cytometric analysis of short and long—term average fluorescence intensity of all HOP-92 fluorescent cells. (B) Flow cytometric analysis of short and long—term average fluorescence intensity of high intensity HOP-92 fluorescent cells. (C) Flow cytometric analysis for short and long—term expression of the average number of all HOP-92 fluorescent cells. (D) Flow cytometric analysis for short and long—term expression of the average number of high intensity HOP-92 fluorescent cells. For each day the measurements were normalized to the value measured for the pRc/CMV expressing *EGFP* vector at the corresponding day. Data shown are the average of 7 short-term independent normalized experiments, 5 long-term independent normalized experiments of all *EGFP*-expressing cells and 4 long-term independent normalized experiments of high *EGFP*-expressing cells (see supporting methods for an explanation). Error bars represent SEM. Statistical comparisons between the promoters were done using the Kruskal—Wallis test with pairwise comparisons. * p ≤0.05, ▲ p ≤0.01(Black- compared to pRc/CMV, Red- compared to SCP2, Green- compared to natural CMV).
(PDF)

S6 Fig Assessment of primers’ quality by qPCR total DNA purified from transiently trasnfected HeLa S3 and SH-SY5Y cells. HeLa S3 and SH-SY5Y cells were transiently transfected with the SCP3 vector expressing *EGFP*, and harvested 4 days post-transfection (P.T.). Total DNA was purified from cells and subjected to qPCR analysis with primers for the GAPDH, *EGFP* and Neomycin genes.
(PDF)

S1 File Supporting Information—Methods. (PDF)

S1 Table Real-Time quantitative PCR of purified transiently transfected plasmid DNA in HeLa S3 and SH-SY5Y cells. HeLa S3 and SH-SY5Y cells were transiently transfected with pRc/CMV, natural CMV, SCP2 or SCP3 vector expressing *EGFP*, and harvested on days 2, 4, 6, 8 and 14 post-transfection (P.T.). Plasmid DNA was purified from cells and subjected to qPCR analysis with primers for the GAPDH, *EGFP* and Neomycin genes. Data shown are the averaged (±SEM) Ct values of 3 independent experiments (each performed in triplicates).
(PDF)
