# Correction: Engineered Promoters for Potent Transient Overexpression

**DOI:** 10.1371/journal.pone.0152449

**Published:** 2016-03-23

**Authors:** The PLOS ONE Staff

Table 1 has been corrected for improved readability. The publisher apologizes for the error. Please see the complete, corrected Table 1 here.

**Table:** 

	−36. . . . . . . . . . . . . . . . . . . . . . . . . . . . . . . .+1. . . . . . . . . . . . . . . . . . . . . . . . . . . . . . . . . . . . . . . . .+45
pRc/CMV	AGGTC**TATATAAG**CAGAGCTCTCTGGCTAACTAGAGAACCCACTGCTTAACTGGCTTATCGAAAT
natural CMV	AGGTC**TATATAAG**CAGAGCTCGTTTAGTGAACCG**TCAGAT**CGCCTGGAGACGCCATCCACGCTGTTTTGACCTCCATAGAA
SCP2	AGGTC**TATATAAG**CAGAGCTCGTTTAGTGAACCG**TCAGAT**CGCCTGGAGACGT**CGAGCCGAGTGGTTGT**GCCTCCATAGAA
SCP3	AGGTC**TATATAAG**CAGAGCTCGTTTAGTGAACCG**TCAG** **TC**CGCCTGGAGACCT**CGAGCCGAGTGGT** **C** **GT**GCCTCCATAGAA
	. . . . .TATA-box. . . . . . . . . . . . . . . . . . . . . .Inr. . . . . . . . . . . . . . . . . .MTE. . . . .DPE. . . . . . . . . . . . . .

## References

[1] Even DY, Kedmi A, Basch-Barzilay S, Ideses D, Tikotzki R, Shir-Shapira H, et al (2016) Engineered Promoters for Potent Transient Overexpression. PLoS ONE 11(2): e0148918 doi:10.1371/journal.pone.0148918 26872062 10.1371/journal.pone.0148918 PMC4752495

