# Correction: Correction: Engineered Promoters for Potent Transient Overexpression

**DOI:** 10.1371/journal.pone.0156345

**Published:** 2016-05-20

**Authors:** The PLOS ONE Staff

## Notice of Republication

This Correction was republished on May 10, 2016, to correct formatting errors in Table 1 that were introduced during the typesetting process. The publisher apologizes for the errors. Please download this article again to view the correct version.
