# Supplementary material for: Engineered Promoters for Potent Transient Overexpression
Source: PLoS One. 2016 Feb 12;11(2):e0148918. doi: 10.1371/journal.pone.0148918 (PMC4752495; doi:10.1371/journal.pone.0148918)
Supplement: S1 Fig — The EGFP gene was cloned into the HindIII and XbaI sites of the commercial pRc/CMV plasmid (Life Technologies). The CMV enhancer was amplified using PCR and the promoters of the natural CMV, SCP2 and SCP3 were cloned into the vector by “dropping in” annealed oligonucleotides and preserving the natural context of the natural CMV enhancer and promoter (i.e., no restriction sites or any artificial spacers were introduced between the enhancer and the promoters). Sequences derived from CMV are shaded in gray; artificial pRc/CMV vector sequence is colored white; MTE and DPE sequences are shaded in yellow. Red lines represent nucleotide changes from SCP2 to SCP3. (PDF) [file pone.0148918.s001.pdf]

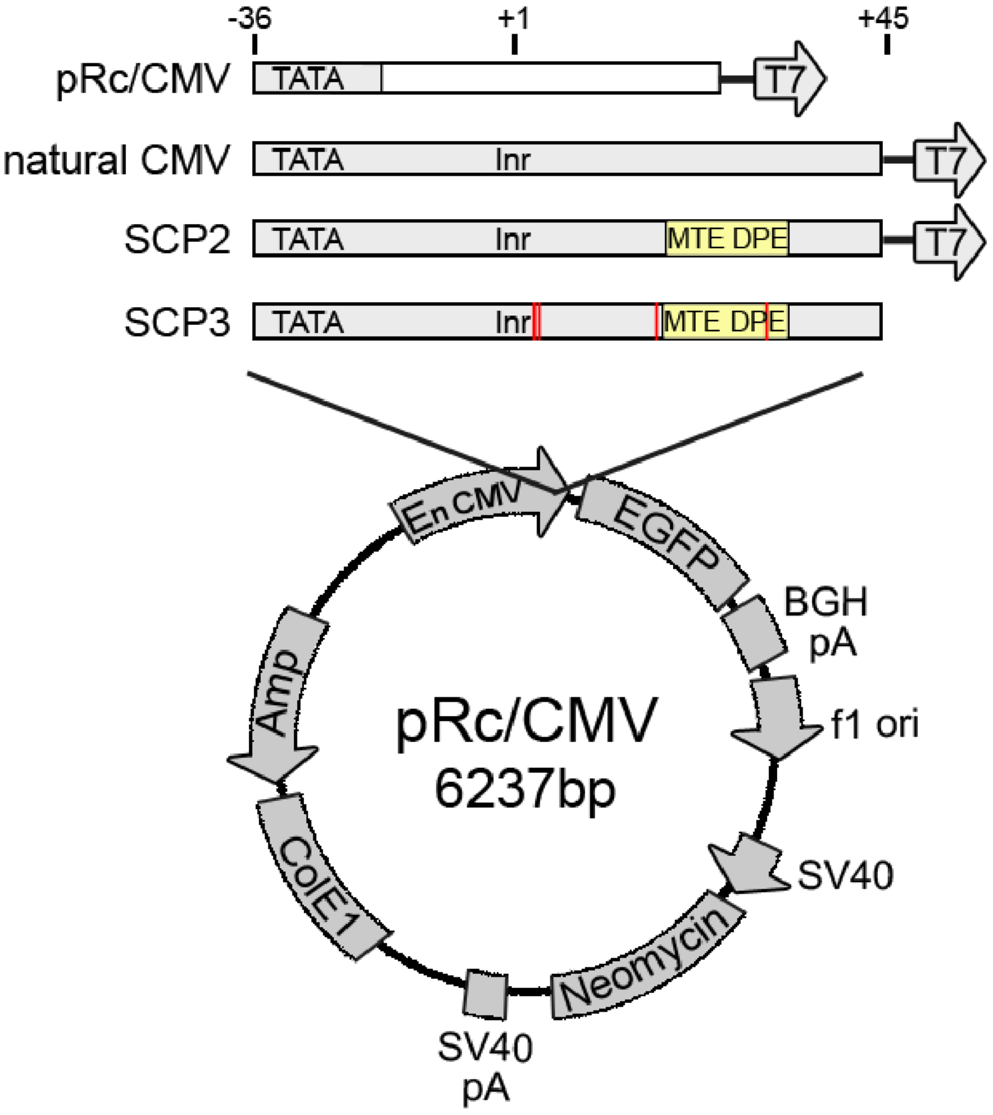

S1 Fig. Schematic illustration of the constructed *EGFP* expression plasmids driven by the various core promoters linked to the CMV enhancer.
