# Supplementary material for: Engineered Promoters for Potent Transient Overexpression
Source: PLoS One. 2016 Feb 12;11(2):e0148918. doi: 10.1371/journal.pone.0148918 (PMC4752495; doi:10.1371/journal.pone.0148918)
Supplement: S2 Fig — SH-SY5Y cells were transiently transfected with either pRc/CMV, natural CMV, SCP2 or SCP3 vector expressing EGFP. The cells were imaged over a 31 days period post-transfection (P.T.). Each circle displays the whole well image constructed by stitching individual microscopic fields. Data shown are representative of 2 independent experiments. (PDF) [file pone.0148918.s002.pdf]

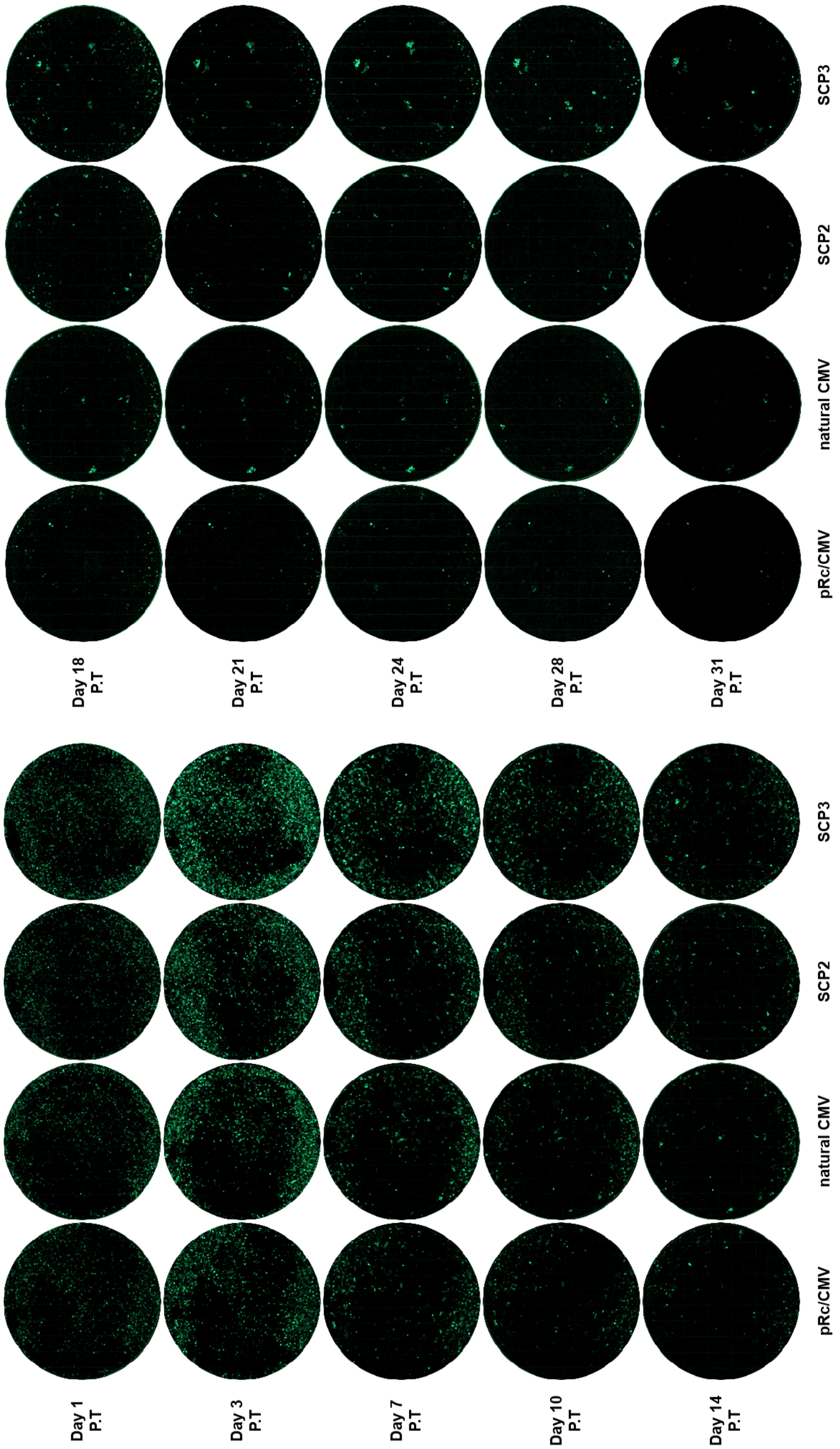

S2 Fig. Live cell imaging of SH-SY5Y cells expressing *EGFP* that is driven by pRc/CMV-based constructs for over 30 days.
