# Supplementary material for: Engineered Promoters for Potent Transient Overexpression
Source: PLoS One. 2016 Feb 12;11(2):e0148918. doi: 10.1371/journal.pone.0148918 (PMC4752495; doi:10.1371/journal.pone.0148918)
Supplement: S4 Fig — HeLa S3 and SH-SY5Y cells were transiently transfected with pRc/CMV, natural CMV, SCP2 or SCP3 vector expressing EGFP. The cells were collected 4–8 days post-transfection (P.T.) for flow cytometric analysis. (A) Flow cytometric analysis of average fluorescence intensity of all HeLa S3 fluorescent cells and SH-SY5Y fluorescent cells. (B) Flow cytometric analysis of average fluorescence intensity of high intensity HeLa S3 fluorescent cells and SH-SY5Y fluorescent cells. (C) Flow cytometric analysis of the average number of all HeLa S3 fluorescent cells and SH-SY5Y fluorescent cells. (D) Flow cytometric analysis of the average number of high intensity HeLa S3 fluorescent cells and fluorescent SH-SY5Y cells. For each day the measurements were normalized to the value measured for the pRc/CMV expressing EGFP vector at the corresponding day. Data shown are the average of 6 independent normalized experiments of all EGFP-expressing cells and 3 independent normalized experiments of high EGFP-expressing cells using HeLa S3 cells (see supporting methods for an explanation) and of 5 independent normalized experiments using SH-SY5Y cells. Error bars represent SEM. Statistical comparisons between the promoters were done using the Kruskal—Wallis test with pairwise comparisons. * p ≤0.05, ▲ p ≤0.01(compared to pRc/CMV). (PDF) [file pone.0148918.s004.pdf]

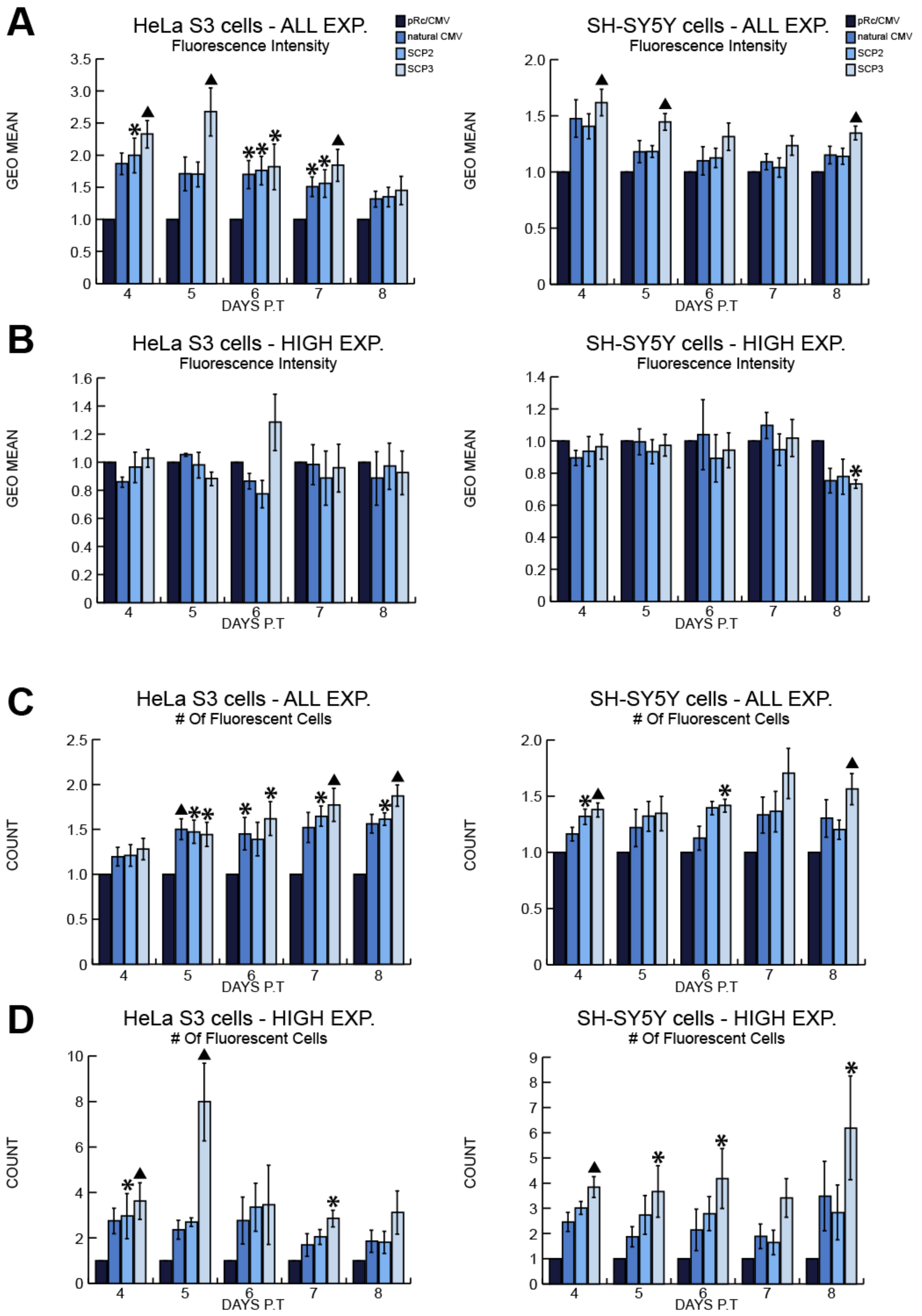

S4 Fig. FACS analysis of long-term average fluorescence intensity and average number of fluorescent HeLa S3 and SH-SY5Y cells.
