# Supplementary material for: Engineered Promoters for Potent Transient Overexpression
Source: PLoS One. 2016 Feb 12;11(2):e0148918. doi: 10.1371/journal.pone.0148918 (PMC4752495; doi:10.1371/journal.pone.0148918)
Supplement: S5 Fig — HOP-92 cells were transiently transfected with pRc/CMV, natural CMV, SCP2 or SCP3 vector expressing EGFP. The cells were collected 1–4 and 4–8 days post-transfection (P.T.) for flow cytometric analysis. (A) Flow cytometric analysis of short and long—term average fluorescence intensity of all HOP-92 fluorescent cells. (B) Flow cytometric analysis of short and long—term average fluorescence intensity of high intensity HOP-92 fluorescent cells. (C) Flow cytometric analysis for short and long—term expression of the average number of all HOP-92 fluorescent cells. (D) Flow cytometric analysis for short and long—term expression of the average number of high intensity HOP-92 fluorescent cells. For each day the measurements were normalized to the value measured for the pRc/CMV expressing EGFP vector at the corresponding day. Data shown are the average of 7 short-term independent normalized experiments, 5 long-term independent normalized experiments of all EGFP-expressing cells and 4 long-term independent normalized experiments of high EGFP-expressing cells (see supporting methods for an explanation). Error bars represent SEM. Statistical comparisons between the promoters were done using the Kruskal—Wallis test with pairwise comparisons. * p ≤0.05, ▲ p ≤0.01(Black- compared to pRc/CMV, Red- compared to SCP2, Green- compared to natural CMV). (PDF) [file pone.0148918.s005.pdf]

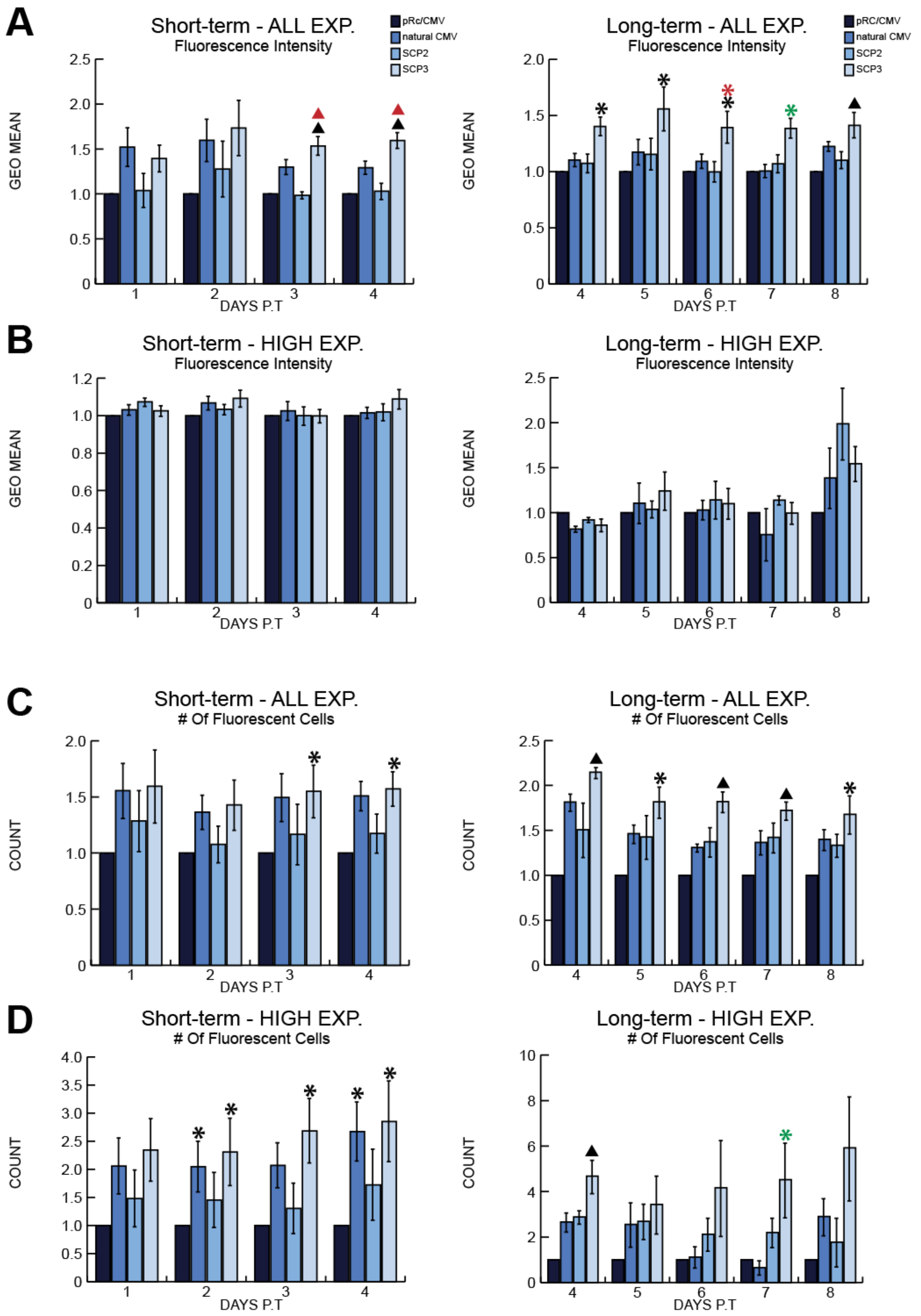

S5 Fig. FACS analysis of short and long -term average fluorescence intensity and average number of fluorescent HOP-92 cells.
