# Supplementary figures and images for: Engineered Promoters for Potent Transient Overexpression
Source: PLoS One. 2016 Feb 12;11(2):e0148918. doi: 10.1371/journal.pone.0148918 (PMC4752495; doi:10.1371/journal.pone.0148918)

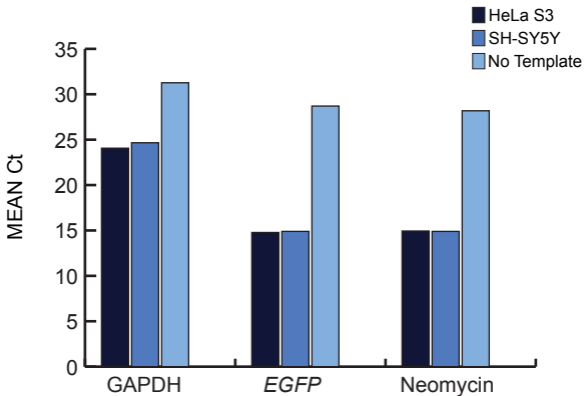

Supplement: S6 Fig — HeLa S3 and SH-SY5Y cells were transiently transfected with the SCP3 vector expressing EGFP, and harvested 4 days post-transfection (P.T.). Total DNA was purified from cells and subjected to qPCR analysis with primers for the GAPDH, EGFP and Neomycin genes. (PDF) [file pone.0148918.s006.pdf]
