# Supplementary material for: Engineered Promoters for Potent Transient Overexpression
Source: PLoS One. 2016 Feb 12;11(2):e0148918. doi: 10.1371/journal.pone.0148918 (PMC4752495; doi:10.1371/journal.pone.0148918)
Supplement: S1 File — (PDF) [file pone.0148918.s007.pdf]

Supporting Information - Methods

Engineered promoters for potent transient overexpression

Dan Y. Even[1¶], Adi Kedmi[1¶], Shani Basch-Barzilay[1¶], Diana Ideses[1], Ravid Tikotzki[1], Hila Shir-Shapira[1], Orit Shefi[2], Tamar Juven-Gershon[1*]

[1] The Mina and Everard Goodman Faculty of Life Sciences, Bar-Ilan University, Ramat-Gan, 5290002, Israel

[2] Faculty of Engineering and Bar-Ilan Institute of Nanotechnologies and Advanced Materials, Bar-Ilan University, Ramat-Gan 5290002, Israel

* Corresponding author:

E-mail: tamar.gershon@biu.ac.il (TJ-G)

[¶]These authors contributed equally to this work

Construction of *EGFP* expression plasmids driven by the various promoters

Construction of the pRc/CMV *EGFP* expression vector

The pRc/CMV expression vector (Life Technologies) contains the CMV enhancer and TATA box but lacks some CMV sequences (including the Inr) that are downstream of the -16 relative to the +1 start site.

The *EGFP* reporter gene was digested out of the pEGFP-N1 vector (Clonetech) using HindIII and XbaI and cloned into pRc/CMV expression vector (Life Technologies), which was digested with HindIII and XbaI.

Construction of the natural CMV *EGFP* expression vector

The natural CMV-driven expression plasmid (used in Juven-Gershon et al. 2006) was constructed in several steps. First, a DNA fragment encompassing the CMV enhancer and an upstream part of the CMV promoter was PCR amplified using the forward primer 5' ATACGCGTTGACATTGATTATTGACT 3' and reverse primer
5' ACGAGCTCTGCTTATATAGACCTCCCAC 3'. The PCR product was cloned into the pGEM-T vector (Promega) and digested with MluI (at the 5' of the CMV enhancer, at 228 relative to the commercial pRc/CMV vector sequence) and SacI (at -16 relative to the TSS of the CMV promoter). In parallel, T4 PNK was used to phosphorylate the 5' ends of the two oligonucleotides. The two oligonucleotides were then annealed to form a dsDNA that encompass from -16 of the CMV to the +45 of the natural CMV sequence (Table 1) that was followed by a T7 promoter and had protruding ends for SacI and HindIII. The PCR product that was digested with MluI and SacI and the phosphorylated dsDNA from SacI to HindIII were ligated (3-way) to the pRc/CMV vector that was digested with MluI and HindIII. Notably, the pRc/CMV contains a T7 promoter just upstream of the HindIII site of the MCS. By digesting the pRc/CMV vector with MluI and HindIII, the T7 promoter is removed from the vector but it is re-introduced as it is included in the annealed oligonucleotides.

The *EGFP* reporter gene was digested out of the pEGFP-N1 vector (Clonetech) using HindIII and XbaI and cloned into this natural CMV expression vector, which was digested with HindIII and XbaI.

Construction of the SCP2 *EGFP* expression vector

The SCP2 driven expression plasmid (used in Juven-Gershon et al. 2006) was constructed in several steps. First, a DNA fragment encompassing the CMV enhancer and an upstream part of the CMV promoter was PCR amplified, cloned into pGEM-T and digested with MluI (at the 5' of the CMV enhancer, at 228 relative to the commercial pRc/CMV vector sequence) and SacI (at -16 relative to the TSS of the CMV promoter), as above. In parallel, we used T4 PNK to phosphorylate the 5' ends of two oligonucleotides and annealed them to form a dsDNA that encompass from -16 of the CMV to the +45 of the designed SCP2 sequence (Table 1) that was followed by a T7 promoter and had protruding ends for SacI and HindIII. The PCR product that was digested with MluI and SacI and the phosphorylated dsDNA with protruding ends for SacI to HindIII were ligated (3-way) to the pRC/CMV vector that was digested with MluI and HindIII. Notably, the pRc/CMV contains a T7 promoter just upstream of the HindIII site of the MCS. By digesting the pRc/CMV vector with MluI and HindIII, the T7 promoter is removed from the vector but it is re-introduced as it is included in the annealed oligonucleotides.

The *EGFP* reporter gene was digested out of the pEGFP-N1 vector (Clonetech) using HindIII and XbaI and cloned into this SCP2 expression vector, which was digested with HindIII and XbaI.

Construction of the SCP3 *EGFP* expression vector

The SCP3 driven expression plasmid was constructed in several steps. First, a DNA fragment encompassing the CMV enhancer and an upstream part of the CMV promoter was PCR amplified, cloned into pGEM-T and digested with MluI (at the 5' of the CMV enhancer, at 228 relative to the commercial pRc/CMV vector sequence) and SacI (at -16 relative to the TSS of the CMV promoter), as above. In parallel, we used T4 PNK to phosphorylate the 5' ends of two oligonucleotides and annealed them to form a dsDNA that encompass from -16 of the CMV to the +45 of the designed SCP3 sequence (Table 1) with protruding ends for SacI and HindIII. The PCR product that was digested with MluI and SacI and the phosphorylated dsDNA from SacI to HindIII were ligated (3-way) to the pRC/CMV vector that was digested with MluI and HindIII. By digesting the pRc/CMV vector with the HindIII site that is located in the MCS of pRc/CMV, just downstream of the T7 promoter, the T7 promoter is removed from the vector.

The *EGFP* reporter gene was digested out of the pEGFP-N1 vector (Clonetech) using HindIII and XbaI and cloned into this SCP3 expression vector, which was digested with HindIII and XbaI.

Cell culture and transfection

HOP-92 cells were cultured in RPMI-1640 supplemented with 10% FBS and grown at 37°C with 5% CO_2. For flow cytometric analysis, HOP-92 cells were plated in 24-well plates one day prior to transfection. Cells were transfected with the various promoter–*EGFP* constructs by using the TransFast reagent (Promega) according to the manufacturer's instructions. Cells were transfected with 0.75µg of each of the different promoter–*EGFP* constructs.

Flow cytometry

Flow cytometric analyses for short- and long-term experiments were performed separately (1-4 and 4-8 days post-transfection, respectively, so that day 4 is common to both analyses), to prevent cell overcrowding. HeLa S3 cells were plated at 2-7 x 10^4 cells per well, SH-SY5Y cells were plated at 3-9 x 10^4 cells per well and HOP-92 were plated at 3-8 x 10^4 cells per well (depending whether the cells were to be analyzed by flow cytometry in days 1-4 or 4-8) in 24-well plates one day prior to transfection. Cells were transfected as described and incubated for 1-8 days. At the indicated time points, cells were harvested, centrifuged at 1000 rpm, resuspended in 0.3 mL PBS and subjected to flow cytometric analysis (Gallios, Beckman Coulter). The fluorescence of *EGFP* was measured in 10,000 cells per sample. The data was analyzed using the FlowJo software. The fluorescence intensity and number of cells expressing *EGFP* for each promoter in each day and each experiment were normalized to the pRc/CMV data obtained in the corresponding experiment (by division of the obtained absolute value by that of the pRc/CMV promoter). The normalized data of each experiment were averaged, and reported in S2 and S3 Figs, where the error bars represent the SEM of all independent normalized experiments. Since in some of the experiments there were no cells that express high fluorescence levels under the regulation of the reference pRc/CMV core promoter, it was

impossible to normalize those individual experiments and therefore they were not averaged, yielding a different number of analyzed experiments regarding the HIGH EXP analyses.
