# Supplementary material for: Engineered Promoters for Potent Transient Overexpression
Source: PLoS One. 2016 Feb 12;11(2):e0148918. doi: 10.1371/journal.pone.0148918 (PMC4752495; doi:10.1371/journal.pone.0148918)
Supplement: S1 Table — HeLa S3 and SH-SY5Y cells were transiently transfected with pRc/CMV, natural CMV, SCP2 or SCP3 vector expressing EGFP, and harvested on days 2, 4, 6, 8 and 14 post-transfection (P.T.). Plasmid DNA was purified from cells and subjected to qPCR analysis with primers for the GAPDH, EGFP and Neomycin genes. Data shown are the averaged (±SEM) Ct values of 3 independent experiments (each performed in triplicates). (PDF) [file pone.0148918.s008.pdf]

Table S1

HeLa S3

		Mean Ct				
Sample	Target	Day 2	Day 4	Day 6	Day 8	Day 14
CMV	GAPDH	32.08 ± 0.12	31.21 ± 0.52	31.03 ± 0.45	31.36 ± 0.18	30.57 ± 0.83
NAT	GAPDH	31.94 ± 0.17	30.78 ± 0.61	30.79 ± 0.55	31.04 ± 0.04	30.41 ± 1.11
SCP2	GAPDH	32.1 ± 0.08	31.1 ± 0.49	31.05 ± 0.73	31.06 ± 0.04	30.89 ± 0.83
SCP3	GAPDH	31.71 ± 0.19	31.04 ± 0.55	30.37 ± 0.73	30.42 ± 0.24	30.4 ± 1.05
No Template	GAPDH	32.29 ± 0.12	32.68 ± 0.26	33.36 ± 0.37	32.22 ± 0.51	33.22 ± 0.15
CMV	EGFP	20.42 ± 0.88	21.2 ± 0.22	21.21 ± 0.23	22.46 ± 0.08	22.06 ± 0.63
NAT	EGFP	20.2 ± 0.67	21.13 ± 0.14	21.63 ± 0.17	22.6 ± 0.25	22.27 ± 0.45
SCP2	EGFP	19.82 ± 0.9	20.59 ± 0.15	21.34 ± 0.12	22.27 ± 0.23	22.19 ± 0.35
SCP3	EGFP	20.29 ± 0.6	20.85 ± 0.07	21.49 ± 0.22	22.74 ± 0.34	22.12 ± 0.42
No Template	EGFP	27.58 ± 0.11	29.03 ± 0.33	28.92 ± 0.15	29.72 ± 0.17	29.5 ± 0.4
CMV	Neo	20.59 ± 0.96	21.44 ± 0.32	21.36 ± 0.19	22.44 ± 0.21	22.18 ± 0.74
NAT	Neo	20.4 ± 0.72	21.4 ± 0.25	21.81 ± 0.15	22.67 ± 0.32	22.58 ± 0.56
SCP2	Neo	19.99 ± 1	20.81 ± 0.24	21.49 ± 0.09	22.37 ± 0.32	22.38 ± 0.53
SCP3	Neo	20.42 ± 0.7	21.08 ± 0.14	21.6 ± 0.22	22.86 ± 0.3	22.29 ± 0.62
No Template	Neo	28.1 ± 1.03	29.13 ± 0.83	28.18 ± 0.23	27.54 ± 0.46	29.4 ± 1.2

SH-SY5Y

		Mean Ct				
Sample	Target	Day 2	Day 4	Day 6	Day 8	Day 14
CMV	GAPDH	31.47 ± 0.31	31.09 ± 0.46	30.07 ± 0.34	29.57 ± 0.57	30.41 ± 1.05
NAT	GAPDH	30.9 ± 0.38	30.96 ± 0.62	29.58 ± 0.34	29.48 ± 0.63	30.23 ± 1.11
SCP2	GAPDH	31.25 ± 0.28	30.99 ± 0.62	29.81 ± 0.12	30.43 ± 0.25	30.34 ± 1.26
SCP3	GAPDH	30.87 ± 0.48	31.16 ± 0.64	29.36 ± 0.22	29.68 ± 0.33	29.7 ± 1.09
No Template	GAPDH	31.9 ± 0.08	32.69 ± 0.14	32.29 ± 0.35	32.12 ± 0.64	32.67 ± 0.38
CMV	EGFP	20.78 ± 0.37	20.64 ± 0.26	21.83 ± 0.57	21.9 ± 0.19	24.17 ± 0.58
NAT	EGFP	19.68 ± 0.16	20.51 ± 0.26	21.21 ± 0.64	21.39 ± 0.14	23.95 ± 0.55
SCP2	EGFP	19.42 ± 0.28	19.85 ± 0.21	21.08 ± 0.68	21.24 ± 0.32	23.48 ± 0.56
SCP3	EGFP	19.73 ± 0.27	20.14 ± 0.23	21.36 ± 0.7	21.71 ± 0.11	23.94 ± 0.61
No Template	EGFP	27.94 ± 0.22	29.04 ± 0.34	28.99 ± 0.18	29.85 ± 0.25	29.71 ± 0.39
CMV	Neo	20.92 ± 0.42	20.95 ± 0.32	22.01 ± 0.52	21.92 ± 0.05	24.36 ± 0.72
NAT	Neo	19.76 ± 0.22	20.7 ± 0.33	21.33 ± 0.61	21.47 ± 0.07	24.17 ± 0.77
SCP2	Neo	19.7 ± 0.37	20.14 ± 0.24	21.3 ± 0.64	21.31 ± 0.28	23.8 ± 0.81
SCP3	Neo	19.92 ± 0.32	20.2 ± 0.24	21.49 ± 0.68	21.74 ± 0.19	24.1 ± 0.79
No Template	Neo	28.32 ± 1.06	28.95 ± 0.8	28.03 ± 0.11	27.75 ± 0.47	28.97 ± 0.88
